# FXI and FXII in experimental stroke and FXI in clinical stroke trials

**DOI:** 10.1186/s42466-026-00540-1

**Published:** 2026-10-16

**Authors:** Stine Mencl, Friederike Langhauser, Jordi Kühne Escolà, Benedikt Frank, Martin Köhrmann, Christoph Kleinschnitz

**Affiliations:** 1 Department of Neurology, University Hospital Essen, Essen, Germany; 2 Center for Translational Neuro- and Behavioral Sciences (C-TNBS), University Hospital Essen, Hufelandstr. 55 Essen, 45147 Germany

**Keywords:** Factor XI, Factor XII, Experimental stroke, Clinical stroke trial

## Abstract

Targeting the contact activation system through Factor XI (FXI) or Factor XII (FXII) inhibition represents a major paradigm shift in antithrombotic therapy for ischemic stroke. Unlike traditional anticoagulants, contact pathway factors drive pathological thrombus propagation and thromboinflammation; crucially, while FXII plays a negligible role in physiological hemostasis, FXI contributes only secondarily, thereby substantially uncoupling antithrombotic efficacy from major bleeding risk. However, a notable disconnect exists between experimental models and clinical development. Preclinical stroke literature, primarily rodent middle cerebral artery occlusion (MCAO) models, is overwhelmingly concentrated on FXII targeting, where FXII deficiency or blockade consistently reduces cerebral infarct volumes, preserves microvascular perfusion, and attenuates thromboinflammation, whereas experimental studies of direct FXI treatment remain sparse. Conversely, human clinical translation has focused largely on oral FXIa inhibitors, maintaining a favorable bleeding profile across indications but yielding mixed efficacy, ranging from neutral Phase II studies and the early termination of OCEANIC-AF to positive secondary stroke prevention data. Following positive OCEANIC-STROKE findings, asundexian (under FDA priority review as of September 2026) emerges as a prospective add-on to antiplatelet therapy for the secondary prevention of noncardioembolic stroke or high-risk TIA, particularly in nonlacunar or atherosclerotic populations. Reconciling preclinical FXII data with clinical FXIa trial outcomes provides a timely rationale to systematically review evidence across MCAO models and human stroke trials to delineate the distinct therapeutic potentials of FXI and FXII inhibition.

## Introduction

Inhibition of coagulation factors XI (FXI) and XII (FXII) has emerged as a promising strategy to uncouple pathological thrombosis from physiological hemostasis [1, 2]. In experimental stroke and thrombus formation studies, targeting FXI and FXII effectively reduces ischemic brain injury while preserving bleeding control [3, 4]. Based on a targeted search of PubMed and ClinicalTrials.gov using specific key words, this review maps the translation of these preclinical concepts into ongoing clinical trials evaluating FXI inhibitors in patients with atrial fibrillation and ischemic stroke. By synthesizing findings from experimental models alongside emerging clinical trial data, this review provides a comprehensive overview of the current landscape and therapeutic potential of FXI and FXII inhibition.

## FXII

### Activation and dual pathways of coagulation factor XII

**Fig. 1:**
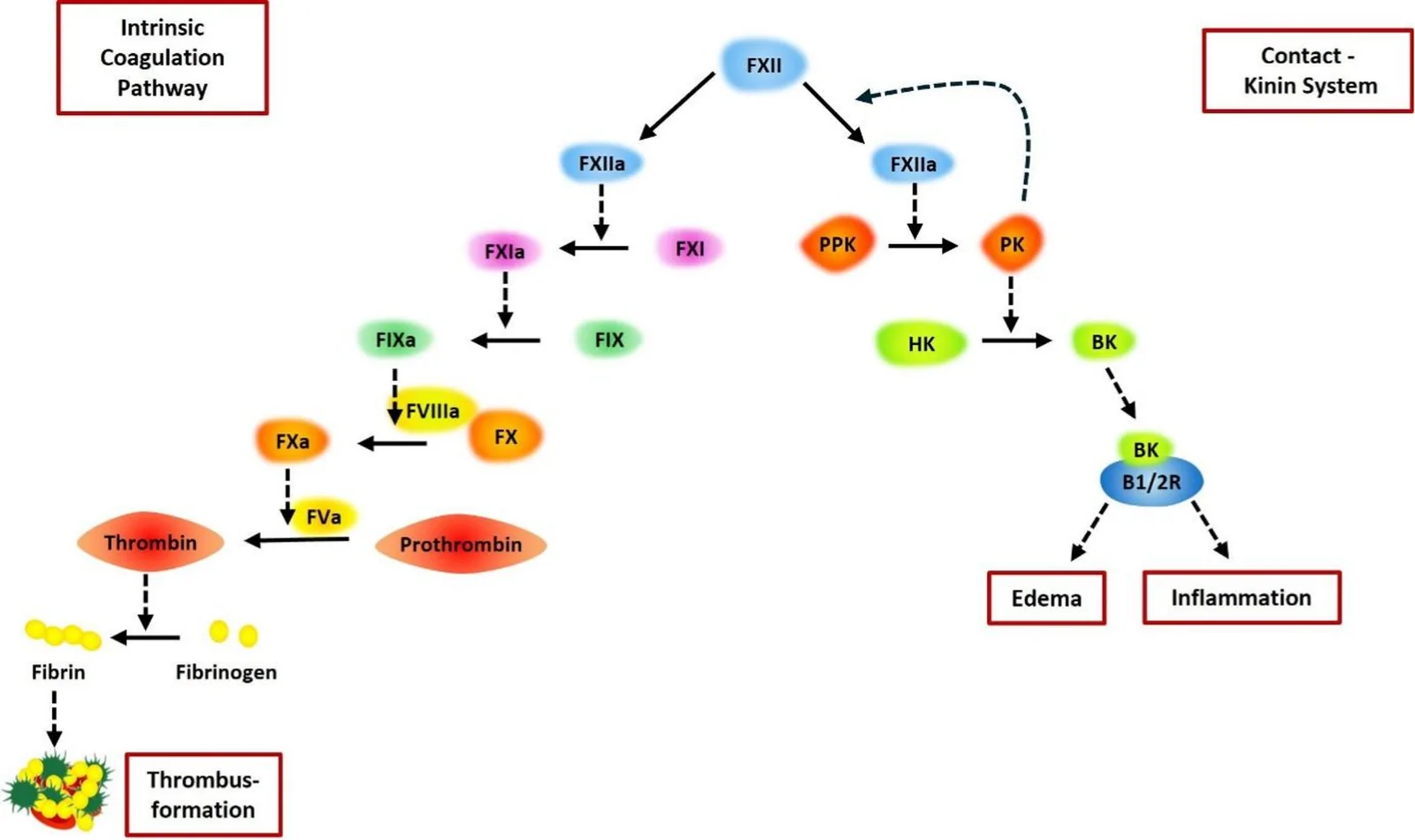
FXII as an initiator of intrinsic coagulation pathway and the contact-kinin-system

Factor XII (FXII) circulates in plasma as an inactive zymogen. Upon contact with negatively charged surfaces, FXII undergoes a conformational change that promotes auto-activation into the active serine protease FXIIa [5]. In biological settings, contact activation is facilitated by inorganic materials, biological polyanionic macromolecules, and nucleic acids. Among these, polyphosphates secreted by activated platelets and neutrophil extracellular traps (NETs) released by neutrophils play a pivotal role in triggering FXII-dependent thrombus formation within cerebral microvessels during ischemic stroke [6]. Once activated, FXIIa initiates two distinct pathophysiological pathways that contribute to ischemic brain damage. FXIIa initiates the intrinsic coagulation pathway by activating FXI, leading to downstream thrombin generation, fibrin accumulation, and pathological intra-arterial thrombus formation (see Fig. 1). Furthermore, FXIIa activates the kallikrein–kinin system by cleaving PPK into active PK. PK functions as an activator FXII by a positive feedback mechanism [7]. In addition, PK processes HK, resulting in the generation of BK. BK binding to endothelial receptors induces the release of pro-inflammatory mediators, including interleukin-1β and interleukin-6, compromises blood-brain barrier (BBB) integrity, and exacerbates hypoxic neural injury [8].

The intrinsic coagulation pathway is initiated by activation of Factor XII (FXII), that cleaves FXI into FXIa, followed by activation of FIX (FIXa). FIXa forms an enzyme complex together with FVIII and activates FX, which along with FVa, stimulates the production of thrombin. Thrombin then converts soluble fibrinogen into insoluble fibrin strands. In addition to its role in triggering thrombus formation via fibrin generation, FXIIa also promotes the activation of the contact-kinin system: FXIIa cleaves plasma prekallikrein (PPK) to form the active serine protease plasma kallikrein (PK), which subsequently cleaves high molecular weight kininogen (HK) to release the inflammatory peptide hormone bradykinin (BK). The binding of BK to its endothelial receptor (bradykinin receptor B1 and B2R) activates signaling cascades that induce two major effects: (1) endothelial cell damage leading to vascular edema and (2) the expression of proinflammatory cytokines.

### Uncoupling hemostasis from thrombosis

A critical challenge in the treatment of stroke is the potential risk of major bleeding complications associated with conventional anticoagulants. In contrast to all other coagulation factors, FXII and FXIIa have no primary role in normal hemostasis. This is based on the observation that individuals with FXII deficiency do not experience abnormal bleeding in response to vascular injury and do not demonstrate prolonged or excessive blood loss during surgical procedures [9]. A meta-analysis also demonstrated an absence of a direct correlation between plasma FXII levels or FXII gene polymorphisms and ischemic stroke risk [10]. This finding suggests a conditional, disease-specific activation mechanism during stroke.

Experimental in vivo studies using knockout mouse models strongly confirm these clinical findings. Like their human counterparts with FXII deficiency, FXII-KO mice display normal development and show no evidence of spontaneous or injury-related bleeding tendencies, although they have significantly prolonged activated partial thromboplastin times (aPTT). When subjected to the transient middle cerebral artery occlusion (MCAO), both FXII-KO and FXI-KO mice display significantly diminished brain infarct volumes compared to wild-type controls. This neuroprotective phenotype is directly accompanied by reduced fibrin accumulation within the ischemic cerebral hemisphere, demonstrating that FXII inhibition provides protection from pathological thrombosis without interfering with physiological hemostasis [4].

### FXII inhibitors in experimental stroke models

A broad spectrum of FXII/FXIIa inhibitors has been investigated in experimental stroke research, ranging from small synthetic molecules to animal-derived peptides, recombinant fusion proteins, and endogenous and recombinant serpins [2]. These are discussed individually below and summarized in Table 1.

#### H-D-Pro-Phe-Arg-chloromethylketone (PCK)

Early pharmacological proof-of-concept was established using H-D-Pro-Phe-Arg-chloromethylketone (PCK), an irreversible inhibitor of FXIIa activity and plasma kallikrein-mediated FXII activation [11]. Mice treated with PCK before MCAO exhibited reduced fibrin formation, decreased intravascular fibrin deposition, and smaller ischemic brain lesions compared to controls without increasing the risk of intracerebral hemorrhage [4].

#### rHA-Infestin-4

Blood-feeding animals have evolved potent antithrombotic mechanisms to facilitate blood feeding, offering a rich source of biological inhibitors. The recombinant fusion protein rHA-Infestin-4, derived from the midgut of Triatoma infestans and fused to recombinant human albumin, specifically inhibits FXIIa and prolongs activated partial thromboplastin time across human, mice, and rat plasma. In a murine model of ischemic stroke, prophylactic injection of rHA-Infestin-4 10 min prior to MCAO reduced infarct volumes to less than 17% of untreated controls and significantly improved both global neurological function and motor function and coordination [12]. Similarly, in rats subjected to MCAO, prophylactic rHA-Infestin-4 reduced infarct size and brain edema and improved neurological function and survival. Therapeutic administration immediately post-reperfusion still yielded improved neurological outcomes and survival, although overall effects were less pronounced [13].

#### Sylvestin

A dual inhibitor of FXIIa and PK purified from the forest leech Haemadipsa sylvestris, it induced a dose-dependent decrease in infarct volumes, which also translated into better functional outcomes, when administered 10 min before or after reperfusion in the murine MCAO model. Furthermore, cerebral blood flow was enhanced and brain fibrinogen deposition, plasma levels of BK, IL-1ß and IL-6 were dose-dependently reduced. Remarkably, Sylvestin showed no signs of a bleeding tendency, failing to significantly prolong bleeding times in skin, cerebral, and tail bleeding assays [14].

#### cycloSD6

Naturally derived and synthetic peptides targeting both FXIIa and PK have shown comparable efficacy. Synthetic modifications have yielded candidates like cycloSD6, a stable cyclic derivative of an immunoglobulin-derived heavy chain peptide sequence (Ser-Leu-Gly-Ala-Ser-Asp). CycloSD6 acts as a dual FXIIa/PK inhibitor, increasing cerebral blood flow, maintaining BBB integrity, and reducing infarct size in both MCAO and photothrombotic stroke models, without increasing bleeding risk [15].

#### Leu-Ser-Glu-Glu-Pro-Glu (LE6)

LE6 is a bat-derived oligopeptide isolated from *Myotis myotis*. The peptide exhibits a dose-dependent inhibition of FXIIa activity, demonstrating a stronger inhibitory potential against FXIIa compared to PK, while showing no hemolytic activity or cytotoxicity. In in vivo models, intravenous administration of LE6 significantly reduced thrombotic clot formation and increased cortical blood flow in mouse brains on day two post-cortical photothrombosis-stroke (CPS) surgery, while also potentially enhancing survival rates. Furthermore, LE6 reduced infarct volumes, attenuated the inflammatory response, and improved BBB integrity following MCAO in a dose dependent manner [16].

#### C1-inhibitor

Endogenous regulatory proteins also demonstrate remarkable neuroprotective utility. C1-inhibitor, a broad-spectrum serpin, simultaneously regulates complement proteases, plasmin, PK, FXIa, and FXIIa [17]. In the context of brain injury, C1 inhibitor exhibits combined anti-inflammatory, anti-edematous, and anti-thrombotic mechanisms. When administered one hour after stroke onset in mice, it halved the infarct size and remained effective up to six hours post-onset. This neuroprotective benefit was preserved in female mice, older cohorts, later stages of infarction, and across species, demonstrating effectiveness in rats as well [18]. A recent study has confirmed the neuroprotective and anti-inflammatory effects of C1 inhibitor in murine brain ischemia/reperfusion injury (MCAO) [19].

### Translational models and clinical outlook

Translating these pre-clinical findings into clinical therapies requires robust testing tools and humanized animal models. The clinical feasibility of FXIIa inhibition in human disease has already been established by garadacimab, a fully human monoclonal antibody targeting FXIIa that demonstrated strong efficacy and safety in Phase III trials for hereditary angioedema prevention [20].

To overcome interspecies differences when testing human-specific targeted therapies like monoclonal antibodies, researchers successfully generated humanized FXII knock-in mice by replacing the murine F12 gene with human F12 [21]. These humanized models retain normal coagulation kinetics and provide an essential translational platform for evaluating novel human FXIIa-specific antithrombotic agents in vivo.

## Conclusion

Targeting Factor XII represents a highly promising therapeutic strategy for ischemic stroke. By simultaneously inhibiting intrinsic clot formation and the inflammatory kallikrein-kinin pathway, FXII targeting attenuates thromboinflammation, maintains BBB integrity, and preserves functional neural tissue without the critical bleeding liabilities associated with standard anticoagulants. Future clinical evaluations leveraging humanized animal models and proven FXIIa inhibitors will clarify the therapeutic window of FXII targeting human ischemic stroke patients.

### Function of Factor XI

First identified in 1953, Factor XI (FXI) deficiency (Hemophilia C) is a rare coagulation disorder with a global prevalence of 1 in 100,000 [22, 23]. The condition stems from reduced plasma FXI levels or impaired catalytic activity of its active form, FXIa. Structurally unique among coagulation factors, FXI arose from a duplication of the KLKB1 gene, sharing structural homology with plasma prekallikrein (PPK). Consequently, nearly all FXI circulates bound to high molecular weight kininogen (HK). Following evolutionary duplication, FXI specialized in coagulation and thrombin generation, whereas PK retained its primary role in kinin generation [24].

FXIa functions primarily in hemostasis, where platelets enhance its overall activity and its rate of Factor IX activation. However, its catalytic domain cleaves many additional substrates, including FXII, HK, FX, FV, FVIII, and FXI itself [25–30].

Beyond coagulation, FXIa influences inflammatory and immune responses. It promotes vascular permeability and inflammation by generating the vasoactive peptide bradykinin (BK), both directly via HK cleavage and indirectly via FXII activation, while also interacting with the complement system. To preserve vessel integrity, FXIa activity is strictly regulated by plasma serpins, including antithrombin, C1-inhibitor, α1-antitrypsin, and protease nexins 1 and 2 [22].

Clinically, FXI plasma levels do not reliably correlate with bleeding severity, complicating risk assessment following trauma, surgery, or childbirth. Substrate interactions offer potential diagnostic clarity. For instance, FXIa normally inhibits tissue factor pathway inhibitor (TFPI); consequently, FXI deficiency combined with high TFPI levels significantly elevates bleeding risk. Understanding these complex substrate relationships is essential for identifying predictive biomarkers of bleeding and safely designing FXIa active-site inhibitors as cardiovascular therapeutics [22].

Although preclinical validation of factor XI (FXI) inhibition in experimental stroke models remains remarkably limited, therapeutic FXI-targeting strategies rapidly advanced into early- and late-phase human clinical trials.

### FXI in experimental thrombosis and stroke

All known FXI inhibitors investigated in experimental animal studies are discussed individually below and summarized in Table 1.

#### Polyclonal Anti-FXI-Antibodies

In a baboon arteriovenous shunt model, Gruber and Hanson [31] demonstrated that polyclonal anti-FXI-IgG selectively halts thrombus propagation while leaving thrombus initiation intact. Although the antibody did not prevent initial clot assembly directly on graft surfaces, it markedly suppressed intraluminal clot growth into the vessel lumen, preventing complete vessel occlusion. This mechanism indicates that FXI sustains the downstream thrombin generation required for a thrombus to expand away from the primary vascular injury site. Ultimately, anti-FXI therapy achieved antithrombotic efficacy comparable to unfractionated heparin without prolonging bleeding time or impairing primary hemostasis.

#### Monoclonal anti-FXI-antibodies

In primate thrombosis models, monoclonal aFXI-antibodies demonstrate FXI’s role in downstream microvascular thrombosis. By blocking FXI activation under shear flow, they prevent platelet activation, stop microaggregate formation, and reduce platelet consumption distal to the primary thrombus site, preventing microvascular downstream occlusion [32, 33].

Specifically targeting contact-mediated FXI activation, the monoclonal mouse antibody 14E11 (analog to gruticibart in humans) disrupts the interaction between FXI and HK. In experimental stroke (MCAO), pre-treatment with 14E11 one day pre-stroke improves cortical reperfusion, reduces cerebral fibrin accumulation, decreases neurological deficits, and limits brain infarct size, establishing that FXIIa-dependent FXI activation directly drives ischemic stroke progression [3].

#### FXI- Antisense-Oligonucleotides (ASO)

In arterial and venous thrombosis models in mouse and baboon, FXI-ASO therapy selectively destabilizes propagating thrombi. While small initial thrombi still form at injury sites, FXI knockdown delays their expansion, reduces their density, and enables native blood flow to clear them, confirming FXI’s specific role in thrombus stabilization rather than initiation [34, 35].

#### PN2KPI

Using mice, PN2KPI halts carotid thrombus propagation in arterial injury models, while in MCAO, PN2KPI significantly reduces brain infarct volume and ischemic brain tissue loss. Interesting here was the fact that retaining just 25% − 50% of baseline FXI-activity is sufficient to protect against cerebrovascular stroke [36].

#### Amrecibart and Cenvacibart

These next-generation therapeutics target intrinsic thrombus propagation under flow in a hemodialysis model in cynomolgus monkeys. Amrecibart selectively blocks FXIIa-driven clot expansion while preserving thrombin-dependent hemostasis. In contrast, cenvacibart shuts down both FXIIa activation and the thrombin-FXI feed-forward loop, delivering complete catalytic inhibition of occlusive thrombus growth [37]. Both compounds are now analyzed in human trials.

**Table 1:** FXI/FXII inhibitors in experimental models of thrombus formation or stroke

Class / Compound	Source / Origin	Mechanism of Action	Species	Prophylactic vs. Therapeutic administration	Bleeding assessment	Major Findings in Thrombus formation or Experimental Stroke Models	References
FXII							
C1-Inhibitor (C1-INH)	Endogenous or recombinant serpin	Broad-spectrum inhibitor (FXIIa, PKa, FXIa, C1r, C1s, Plasmin)	Mice, Rats	1 h pro / 0–6 h ther	MRI	Halved infarct volume up to 6 h post-stroke in young/old, male/female mice and rats via combined anti-inflammatory, anti-edematous, and antithrombotic actions.	[17–19]
cycloSD6	Synthetic cyclopeptide (derived from immunoglobulin heavy chain region)	Dual FXIIa and PKa inhibitor	Mice	10 min pro	Mouse tail truncation	High chemical stability; reduced infarct volumes, preserved BBB integrity, improved CBF, and elevated survival in cortical photothrombosis and MCAO models.	[15]
LE6	Bat-derived oligopeptide (*Myotis myotis*)	FXIIa inhibitor (> PKa)	Mice	15 min pro	Mouse tail truncation	Non-cytotoxic, non-hemolytic; reduced thrombus formation, preserved BBB, reduced inflammatory response, restored CBF, and improved motor outcomes in MCAO and photothrombosis models.	[16]
PCK	Synthetic peptide	Irreversible inhibitor of FXIIa amidolytic activity and PKa	Mice	0 min ther	Mouse tail truncation	Reduced ischemic brain damage, intravascular fibrin deposition, and fibrin formation in murine stroke.	[4, 11]
rHA-Infestin-4	Recombinant fusion protein (*Triatoma infestans* midgut fused to human albumin)	Specific FXIIa protease inhibitor	Mice, Rats	70 min pro / 0 min ther	Mouse tail truncation	Prophylactic administration reduced infarct volume, improved Bederson and grip test scores in tMCAO mice; Reduced edema, infarct size, and improved survival in rat MCAO (prophylactic & therapeutic).	[12, 13]
Sylvestin	Bio-active peptide (*Haemadipsa sylvestris* forest leech)	Dual FXIIa and PKa inhibitor	Mice	10 min pro / 10 min ther	Skin, Cerebral, Tail truncation	Reduced thromboinflammation, brain fibrin, bradykinin, IL-1ß, and IL-6. Decreased thrombus formation and increased cerebral blood flow (CBF) post-MCAO without prolonging tail, skin, or brain bleeding.	[14]
FXI							
10C9	anti-FXI antibody (humanized antibody: Abelacimab)	Binds near the FXIa active site, preventing catalytic activity	Baboons	pro	Bleeding time (BT)	Inhibition of FXIa with the FXI-Ab 1A6, 14E11 or 10C9 can regulate platelet function and prevent platelet-driven amplification of circulating thrombi formation in prothrombotic conditions involving direct contact pathway agonists and widespread vascular injury both in vitro and in a nonhuman primate model of thrombus formation	[31, 33]
14E11	Mouse anti-FXI antibody (humanized antibody: gruticibart)	Targets a highly conserved region of the A2 domain and specifically blocks FXIIa-mediated activation of FXI as well as, conversely, FXIa-mediated activation of FXII, in vitro	Mice	24 h pro	Mouse tail truncation; visual examination of brain	Mice that received the antibody before MCAO showed reduced cerebral infarction and fibrin deposition (thrombosis), increased cortical reperfusion, and improved neurological behavior, with no detectable hemostasis impairment.	[3]
aFXI	Monospecific antihuman FXI polyclonal goat antibody	Inhibition of FXI by binding to different sites	Baboons	pro	Bleeding time (BT)	Reduced intraluminal thrombus growth in a nonhuman primate model of thrombus formation	[29, 31]
Amrecibart (REGN9933)	FXI monoclonal antibody	Specifically prevent FXI activity induced by FXIIa	cynomolgus monkeys	pro	Bleeding time (BT)	A hemodialysis model in cynomolgus monkeys show anticoagulant activity without increasing bleeding	[36, 37]
ASO	Synthetic FXI antisense oligonucleotide	Degradation of FXI mRNA	Mice; Baboon	24 h pro	Mouse tail truncation; Hepatectomy surgical; Bleeding time (BT)	FXI ASO treatment produced potent, dose-dependent antithrombotic activity in various venous and arterial thrombosis models in mice and non-human primates; time-dependent reduction of FXI mRNA levels in mice: onset 1d, max. inhibition 2-7d, return to basal 14–28 d; Baboon injection 3x weekly for 39–60 d	[34, 35]
aXIMab (1A6)	Neutralizing anti–human FXI monoclonal antibody	Binds the A3 domain and inhibits FXI activation by FXIIa, and FIX and FV activation by FXIa.	Baboon	24 h pro	Bleeding time (BT)	In a nonhuman primate model of thrombus formation, 1A6 limited platelet and fibrin deposition and prevented occlusion of collagen-coated vascular grafts; abrogate downstream thrombin– antithrombin complex formation and the distal release of the platelet chemokine β-thromboglobulin	[32]
Cenvacibart (REGN7508)	FXI monoclonal antibody	Targets the catalytic domain to completely block FXI activity	cynomolgus monkeys	pro	Bleeding time (BT)	A hemodialysis model in cynomolgus monkeys show anticoagulant activity without increasing bleeding	[36, 37]
PN2KPI	Kunitz protease inhibitor domain of protease nexin-2	Specific FXI inhibitor of the catalytic domain	Mice	45 pro	Mouse tail truncation;	Stops carotid thrombus propagation in arterial injury models; significantly reduces brain infarct volume and ischemic brain tissue loss after MCAO	[36]

aFXI, anti-Factor XI polyclonal antibody; ASO, antisense oligonucleotide; BBB, blood-brain barrier; BT, bleeding time; C1-INH, C1-esterase inhibitor; C1r/C1s, complement C1 subcomponents r and s; CBF, cerebral blood flow; d, day(s); FIX, Factor IX; FV, Factor V; FXI/FXIa, Factor XI / activated Factor XI; FXII/FXIIa, Factor XII / activated Factor XII; h, hour(s); IL-1β, interleukin-1 beta; IL-6, interleukin-6; MCAO, middle cerebral artery occlusion; min, minute(s); MRI, magnetic resonance imaging; mRNA, messenger ribonucleic acid; PCK, D-Phe-Pro-Arg chloromethyl ketone; PKa, plasma kallikrein; PN2KPI, Kunitz protease inhibitor domain of protease nexin-2; pro, prophylactic administration; rHA, recombinant human albumin; ther, therapeutic administration; tMCAO, transient middle cerebral artery occlusion

## FXI in clinical trials

### Introduction

Preventing recurrent ischemic stroke requires a balance between sufficient antithrombotic efficacy and an acceptable risk of bleeding. Because this balance remains imperfect, inhibition of FXI or FXIa has been evaluated in different clinical settings as a novel approach to narrow this gap.

### Therapeutic approaches to factor XI inhibition

Three therapeutic modalities targeting FXI or FXIa have entered clinical development: small molecule inhibitors, monoclonal antibodies and antisense oligonucleotides (ASOs). These differ in molecular target, administration route, onset and duration of action, and potential for rapid treatment interruption [38]. The details known are discussed individually below and summarized in Table 1.

#### Small molecule inhibitors

Asundexian and milvexian are orally administered, direct, reversible inhibitors of FXIa that reach peak plasma concentrations within a few hours. Elimination half-lives are 14–17 h for asundexian (administered once daily) and 8–14 h for milvexian (evaluated in twice-daily regimens in phase III) [39, 40]. Both compounds are predominantly cleared through non-renal pathways. Therefore, potential pharmacokinetic drug interactions should be considered. Milvexian is metabolized predominantly by CYP3A4/5 and is a substrate of P-glycoprotein. Thus, its exposure is substantially affected by strong CYP3A and P-glycoprotein inhibitors or inducers [41, 42]. Asundexian undergoes mainly CES1-mediated hydrolysis, with a smaller contribution of CYP3A4-mediated metabolism. Nevertheless, combined modulation of CYP3A4 and P-glycoprotein can also alter asundexian exposure [43].

#### Monoclonal antibodies

Monoclonal antibodies are administered parenterally and provide rapid, prolonged FXI/FXIa inhibition. The most clinically advanced agent in stroke prevention is abelacimab, which dual-binds FXI and FXIa, locking FXI in an inactive conformation to prevent activation while neutralizing already active FXIa [44]. Intravenous administration results in rapid suppression of free FXI within one hour, whereas monthly subcutaneous administration is used for long-term treatment [45].

#### Antisense Oligonucleotides (ASO)

ASOs, including IONIS-FXI_Rx_ and its ligand-conjugated successor fesomersen, reduce hepatic FXI synthesis over several weeks, producing a gradual, sustained decrease in circulating FXI. Clinical studies have evaluated ASOs in venous thromboembolism and hemodialysis, but no dedicated stroke-prevention trials have been reported.

### Clinical evidence in stroke prevention

The clinical relevance of FXI inhibition depends on whether treatment complements or replaces established antithrombotics. In noncardioembolic stroke, FXIa inhibitors are evaluated as an add-on component to antiplatelet therapy. In atrial fibrillation (AF), development aims to either replace standard oral anticoagulation in eligible patients or provide an alternative for standard oral anticoagulation ineligible patients. Clinical trials from these distinct scenarios are summarized in Table 2.

#### Noncardioembolic ischemic stroke or high-risk TIA

Despite guideline-directed therapy, including short-term dual antiplatelet treatment, patients with noncardioembolic ischemic stroke or high-risk TIA remain at substantial risk of recurrent ischemic events [46–48]. Dual-pathway inhibition combining aspirin with low-dose rivaroxaban in the COMPASS trial reduced major cardiovascular events but increased major bleeding [49, 50]. FXI/FXIa inhibition aims to extend this dual-pathway concept without a clinically meaningful increase in bleeding.

#### Phase II studies

PACIFIC-Stroke and AXIOMATIC-SSP were the first randomized dose-finding trials to evaluate oral FXIa inhibition in patients with recent noncardioembolic stroke or high-risk TIA. Both investigated FXIa inhibition in addition to antiplatelet therapy and used imaging-enriched efficacy endpoints that combined recurrent ischemic stroke with covert brain infarction on magnetic resonance imaging. Bleeding events were the main safety outcome, although their definitions and assessment periods slightly differed.

PACIFIC-Stroke randomized 1,808 patients within 48 h after a noncardioembolic ischemic stroke to asundexian 10, 20, or 50 mg once daily or placebo in addition to single or dual antiplatelet therapy [51]. Asundexian did not demonstrate a dose-dependent reduction in the primary efficacy endpoint at 26 weeks. The primary safety outcome was rare and no statistically significant differences between groups were observed.

AXIOMATIC-SSP randomized 2,366 patients with acute noncardioembolic ischemic stroke or high-risk TIA to five different milvexian regimens or placebo [52]. Participants received aspirin and clopidogrel for the first 21 days, followed by aspirin alone. No clear dose–response relationship was observed for the composite efficacy endpoint. However, symptomatic recurrent stroke was numerically less frequent with most milvexian doses. The main safety outcome, major bleeding at 90 days, was uncommon and no fatal bleeding occurred. However, a modest numerical increase in major bleeding, predominantly of gastrointestinal origin, was observed at milvexian doses of 50 mg twice daily and higher.

Both trials were therefore neutral with respect to their primary efficacy endpoints. Nevertheless, they established the feasibility of combining FXIa inhibition with antiplatelet therapy and provided the safety data required for phase III development. Furthermore, both showed numerical signals for fewer symptomatic ischemic strokes at selected doses. A subsequent analysis of PACIFIC-Stroke suggested that covert brain infarcts are etiologically heterogeneous and may differ from clinically manifest recurrent strokes in their response to antithrombotic treatment [53]. Treatment effects on a composite of covert infarction and symptomatic stroke may therefore not necessarily reflect effects on clinically manifest events.

#### Phase III studies

OCEANIC-STROKE randomized 12,327 patients within 72 h after a noncardioembolic ischemic stroke or high-risk TIA to asundexian 50 mg once daily or placebo, in addition to planned single or dual antiplatelet therapy [54]. For trial inclusion, either a nonlacunar infarct on imaging, established atherosclerotic disease, or cerebrovascular plaque was required. The primary efficacy outcome was ischemic stroke, defined clinically. After a median follow-up of approximately 19 months, ischemic stroke occurred in 6.2% of patients assigned to asundexian and 8.4% assigned to placebo, corresponding to a cause-specific hazard ratio (HR) of 0.74 (95% confidence interval [CI], 0.65–0.84).

OCEANIC-STROKE provides the first evidence that inhibition of FXIa can improve secondary prevention after noncardioembolic ischemic stroke or high-risk TIA. The 26% relative reduction in ischemic stroke, including a reduction in disabling or fatal events, was achieved on top of standard antiplatelet therapy without a statistically significant increase in major bleeding.

Nonetheless, limitations of this study include a selected a population with an enhanced vascular risk and most participants had mild ischemic strokes, whereas high-risk TIA accounted for approximately 5% of index events. The findings may therefore not be directly transferable to patients with lacunar stroke, severe disabling stroke, or those without clinical or imaging evidence of atherosclerotic disease.

#### Ongoing studies

LIBREXIA-STROKE (NCT05702034) is an ongoing phase III study comparing milvexian 25 mg twice daily with placebo in addition to standard antiplatelet therapy after noncardioembolic acute ischemic stroke or high-risk TIA. The trial has completed enrollment of 12,532 participants, with topline results expected in late 2026.

### Patients with AF eligible for standard oral anticoagulation

Direct oral anticoagulants (DOACs) effectively prevent stroke in AF, but bleeding concerns contribute to underuse and complicate emergency management by frequently disqualifying patients presenting with acute ischemic stroke from receiving intravenous thrombolysis (IVT) [55, 56]. FXI or FXIa inhibition has therefore been investigated as a potentially safer anticoagulant strategy, with the aim of maintaining efficacy comparable to that of established oral anticoagulants while further reducing major bleeding.

#### Phase II studies

PACIFIC-AF was a phase II dose-finding study comparing asundexian 20 or 50 mg once daily with standard-dose apixaban in 755 patients with AF [57].

Participants had at least one bleeding risk feature including history of previous bleeding, estimated glomerular filtration rate of 30–50 mL/min, or current indication for aspirin. The trial met its primary endpoint, which was a composite of major and clinically relevant nonmajor bleeding according to ISTH criteria. Event rates were numerically lower with both asundexian doses. Furthermore, FXIa activity was inhibited by up to 94% at peak with the 50 mg dose.

AZALEA-TIMI 71 evaluated the long-acting monoclonal antibody abelacimab in 1,287 patients with AF. Participants were randomized to abelacimab 90 or 150 mg administered subcutaneously once monthly or rivaroxaban 20 mg once daily. Primary end point was major or clinically relevant nonmajor bleeding. The trial was stopped early because both abelacimab regimens markedly reduced major or clinically relevant nonmajor bleeding compared with rivaroxaban [58].

Together, PACIFIC-AF and AZALEA-TIMI 71 supported the hypothesis that FXI inhibition may cause less major bleeding than factor Xa inhibition. However, they were neither designed nor powered to determine whether this safety advantage could be achieved without a clinically relevant reduction in cardioembolic protection.

#### Phase III studies

OCEANIC-AF compared asundexian 50 mg once daily with standard-dose apixaban in patients with AF at increased risk of both thromboembolism and bleeding [59]. The primary efficacy objective was to establish non-inferiority of asundexian for the prevention of stroke or systemic embolism, whereas the primary safety objective was to demonstrate superiority with respect to major bleeding according to ISTH criteria. The intention-to-treat population comprised 14,810 participants. Nearly one-fifth had a previous stroke or transient ischemic attack. The trial was terminated prematurely after a median follow-up of 155 days because of an early and persistent excess of thromboembolic events with asundexian. Stroke or systemic embolism occurred in 98 participants receiving asundexian and 26 receiving apixaban. The difference was predominantly attributable to ischemic stroke, which occurred in 85 vs. 21 participants, respectively. Cardiovascular and all-8ause mortality did not differ significantly between groups. Bleeding outcomes consistently favored asundexian, with major bleeding occurring in 17 participants receiving asundexian and 53 receiving apixaban. Nevertheless, the composite of stroke, systemic embolism, or major bleeding occurred more frequently with asundexian than with apixaban, indicating that the reduction in bleeding did not outweigh the decrease in antithrombotic efficacy.

OCEANIC-AF therefore demonstrated that asundexian 50 mg once daily is not an effective alternative to apixaban in patients with AF eligible for standard anticoagulation. The findings emphasize the importance of adequately powered outcome trials. In this case, insufficient antithrombotic efficacy ultimately led to unfavorable clinical outcomes, despite the favorable bleeding profile and substantial pharmacodynamic inhibition of FXIa in the phase II study.

#### Ongoing studies

LIBREXIA-AF (NCT05757869) is an event-driven, double-blind phase III trial comparing milvexian 100 mg twice daily with guideline-adjusted apixaban in patients with AF or atrial flutter who are eligible for long-term anticoagulation. The primary objective is to assess whether milvexian is non-inferior to apixaban for prevention of stroke or non-central-nervous-system systemic embolism. Major and clinically relevant non-major bleeding are key secondary outcomes. The study has completed enrollment of 20,284 participants and is currently active but no longer recruiting. Topline results are expected in late 2026.

ROXI-ATLAS (NCT07175428) is a three-arm phase II trial comparing monoclonal FXI antibodies REGN7508 and REGN9933 with apixaban. The study is actively recruiting and plans to enroll 1,200 participants with AF and an indication for long-term anticoagulation. Study completion is estimated for April 2027.

ROXI-EVEREST (NCT07626411) is a phase III trial that will compare REGN7508 with apixaban for prevention of stroke and systemic embolism in patients with AF eligible for oral anticoagulation. The study aims to enroll 15,364 participants and study completion is expected of December 2029.

### Patients with AF ineligible for standard oral anticoagulation

The rationale for studying inhibition of FXI of FXIa in patients with AF who are considered unsuitable for standard oral anticoagulation is derived primarily from the lower bleeding rates observed with FXI or FXIa inhibitors when compared to DOAC. Thus, the relevant comparator in this setting is placebo or no anticoagulation. Consequently, an FXI or FXIa inhibitor would not necessarily need to match the antithrombotic efficacy of a DOAC to provide clinical benefit. It would, however, need to prevent a clinically meaningful number of thromboembolic events without causing an unacceptable increase in bleeding. Two phase III programs are currently evaluating this strategy.

LILAC-TIMI 76 (NCT05712200) is an event-driven, randomized, double-blind phase III trial comparing abelacimab 150 mg administered subcutaneously once monthly with placebo. The study plans to enroll approximately 2,500 patients with AF who have been deemed unsuitable for oral anticoagulation or are unwilling to receive it. The primary efficacy endpoint is ischemic stroke or systemic embolism, while major bleeding according to ISTH criteria is the principal safety outcome. The study remains in recruitment, and completion is expected in December 2027.

ROXI-INCLINE (NCT07430956) is a randomized phase III trial evaluating the two monoclonal FXI antibodies REGN7508 and REGN9933 individually against placebo. The planned enrollment is 2,628 participants with AF for whom oral anticoagulation is considered unsuitable. The primary objective is to demonstrate superiority of each antibody over placebo for the prevention of ischemic stroke or systemic embolism. Recruitment began in 2026, and the study is expected to conclude in 2029.

Bayer had planned OCEANIC-AFINA as a placebo-controlled phase III trial of asundexian in approximately 2,000 patients who were considered ineligible for oral anticoagulation because of increased bleeding risk. However, recruitment had not started when OCEANIC-AF was terminated for lack of efficacy and Bayer subsequently announced that the OCEANIC-AFINA design would be re-evaluated.

### Practical considerations and future directions

Across clinical settings, FXI inhibition has shown a generally favorable bleeding profile, although its future role is likely to differ by indication. Following positive OCEANIC-STROKE findings, asundexian, currently under FDA priority review, emerges as a prospective add-on to antiplatelet therapy for secondary noncardioembolic stroke or high-risk TIA prevention, particularly in nonlacunar or atherosclerotic populations. The ongoing LIBREXIA-STROKE trial will determine if this efficacy extends to milvexian as a broader class effect. Broad implementation requires addressing potential pharmacokinetic interactions with enzyme-modulating antiseizure drugs, azole antifungals, macrolides, or antivirals. Managing emergency scenarios remains challenging due to the absence of compound-specific calibrated assays, validated cutoff values, and targeted antidotes [60]. Therefore, clear emergency protocols should be developed before widespread clinical adoption, particularly if FXI inhibition enters routine secondary prevention after noncardioembolic stroke.

By contrast, FXI and FXIa inhibition currently has no established role as a replacement for direct oral anticoagulants in patients with AF who are eligible for standard anticoagulation. OCEANIC-AF demonstrated insufficient antithrombotic efficacy of asundexian 50 mg once daily compared with apixaban. The potential benefits of other agents, longer-acting dosing regimens, or antibody-mediated FXI inhibition, possibly in the subgroup of patients with AF deemed unsuitable for conventional anticoagulation due to a very high risk of bleeding, have yet to be demonstrated prospectively.

**Table 2:** Completed and ongoing clinical trials of factor XI inhibition across stroke-prevention settings

Clinical setting	Trial; phase and status	Treatment and comparator	Population and background therapy	Primary endpoint(s)	Main findings or status
**Noncardioembolic ischemic stroke or high-risk TIA**	PACIFIC-Stroke NCT04304508 Phase IIb; completed	Asundexian 10, 20, or 50 mg once daily vs. placebo	Recent noncardioembolic ischemic stroke; single or dual antiplatelet therapy; *N* = 1,808	Efficacy: symptomatic ischemic stroke or covert brain infarction at 26 weeks. Safety: ISTH major or clinically relevant nonmajor bleeding	No dose-dependent reduction in the primary efficacy endpoint. Bleeding events were uncommon, without a statistically conclusive difference from placebo.
	AXIOMATIC-SSP NCT03766581 Phase II; completed	Milvexian 25 mg once daily or 25, 50, 100, or 200 mg twice daily vs. placebo	Acute noncardioembolic ischemic stroke or high-risk TIA; DAPT for 21 days followed by aspirin; *N* = 2,366	Efficacy: symptomatic ischemic stroke or new covert brain infarction at 90 days. Safety: modified BARC type 3 or 5 bleeding	No clear dose-response for the primary endpoint. Symptomatic stroke was numerically less frequent with most regimens, but not with 200 mg twice daily. Major bleeding was uncommon, with a numerical increase at doses > = 50 mg twice daily.
	OCEANIC-STROKE NCT05686070 Phase III; completed	Asundexian 50 mg once daily vs. placebo	Recent noncardioembolic ischemic stroke or high-risk TIA; single or dual antiplatelet therapy; *N* = 12,327	Efficacy: ischemic stroke. Safety: ISTH major bleeding	Ischemic stroke: 6.2% vs. 8.4% (HR 0.74). Major bleeding: 1.9% vs. 1.7% (HR 1.10).
	LIBREXIA-STROKE NCT05702034 Phase III; active, not recruiting	Milvexian 25 mg twice daily vs. placebo	Acute ischemic stroke or high-risk TIA; standard single or dual antiplatelet therapy; *N* = 12,532 (actual)	Recurrent ischemic stroke; major bleeding	Enrollment completed. Primary completion estimated November 2026; study completion estimated December 2026.
**AF eligible for standard oral anticoagulation**	PACIFIC-AF NCT04218266 Phase IIb; completed	Asundexian 20 or 50 mg once daily vs. apixaban	AF with increased bleeding risk; *N* = 755	ISTH major or clinically relevant nonmajor bleeding	Bleeding was reduced in the pooled asundexian analysis and with 50 mg individually; the result for 20 mg alone was inconclusive. Not powered for thromboembolic efficacy.
	AZALEA-TIMI 71 NCT04755283 Phase IIb; completed	Abelacimab 90 or 150 mg subcutaneously once monthly vs. rivaroxaban	AF with moderate-to-high thromboembolic risk; *N* = 1,287	ISTH major or clinically relevant nonmajor bleeding	Stopped early because of substantially less bleeding with abelacimab. Incidence rates: 3.2 and 2.6 vs. 8.4 events per 100 person-years. Not powered for comparative stroke prevention.
	OCEANIC-AF NCT05643573 Phase III; terminated early	Asundexian 50 mg once daily vs. apixaban	AF with increased thromboembolic risk; *N* = 14,810	Efficacy: stroke or systemic embolism. Safety: ISTH major bleeding	Stroke or systemic embolism: 1.3% vs. 0.4% (HR 3.79). Major bleeding: 0.2% vs. 0.7% (HR 0.32). Terminated because of inferior efficacy.
	LIBREXIA-AF NCT05757869 Phase III; active, not recruiting	Milvexian 100 mg twice daily vs. guideline-adjusted apixaban	AF or atrial flutter eligible for long-term anticoagulation; *N* = 20,284 (actual)	Stroke or non-CNS systemic embolism; major and clinically relevant nonmajor bleeding	Enrollment completed. Primary and final study completion estimated October 2026.
	ROXI-ATLAS NCT07175428 Phase II; recruiting	REGN7508 or REGN9933 vs. apixaban	AF with an indication for long-term anticoagulation; planned *N* = 1,200	Bleeding safety, pharmacokinetics, and pharmacodynamics	Safety and dose-selection study; not designed as a definitive stroke-efficacy trial. Study completion estimated April 2027.
	ROXI-EVEREST NCT07626411 Phase III; not yet recruiting	REGN7508 vs. apixaban	AF eligible for oral anticoagulation; planned *N* = 15,364	Stroke or systemic embolism; bleeding outcomes	Definitive active-comparator efficacy trial. Study completion estimated December 2029.
**AF unsuitable for standard oral anticoagulation**	LILAC-TIMI 76 NCT05712200 Phase III; recruiting	Abelacimab 150 mg subcutaneously once monthly vs. placebo	AF considered unsuitable for available oral anticoagulation; planned *N* = 2,500	Ischemic stroke or systemic embolism; major bleeding	Event-driven placebo-controlled outcome trial. Study completion estimated December 2027.
	ROXI-INCLINE NCT07430956 Phase III; recruiting	REGN7508 or REGN9933 vs. placebo	AF for which oral anticoagulation is considered unsuitable; planned *N* = 2,628	Ischemic stroke or systemic embolism	Each antibody is tested individually against placebo. Primary completion estimated March 2029; study completion estimated June 2029.

Enrollment and completion dates for ongoing trials reflect the ClinicalTrials.gov records available on July 21, 2026. OCEANIC-AFINA was planned but not initiated and is therefore not included

AF, atrial fibrillation; BARC, Bleeding Academic Research Consortium; CNS, central nervous system; DAPT, dual antiplatelet therapy; HR, hazard ratio; ISTH, International Society on Thrombosis and Hemostasis; TIA, transient ischemic attack

## Discussion and conclusion

Although early historical reports noted that the index FXII-deficient patient, John Hageman, died of pulmonary embolism following severe pelvic trauma [61], extensive subsequent epidemiological, genetic, and preclinical studies, as well as clinical trials with therapeutic anti-FXII/FXIIa agents for the prevention of hereditary angioedema, have confirmed that FXII deficiency or targeted FXII inhibition does not cause thrombophilia or a prothrombotic state [2]. Nevertheless, a historical reluctance to translate FXII clinically paved the way for FXI to dominate human trials, albeit on a surprisingly thin foundation of stroke-specific preclinical data.

A critical limitation of the current evidence base is the pronounced asymmetry between preclinical research and clinical translation in Factor XI (FXI) inhibition. Despite the clinical FXI pipeline emerging as one of the most promising novel antithrombotic strategies, direct preclinical evaluation of FXI targeting in acute stroke remains remarkably sparse, with MCAO studies restricted almost exclusively to a few key agents, such as the monoclonal antibody 14E11 and the domain inhibitor PN2KPI [3, 36]. In contrast, the extensive preclinical rationale that propelled FXI inhibitors into large-scale human trials relied heavily on systemic thrombosis, vascular injury, and cardiovascular paradigms. This reliance on general thrombosis models rather than indication-specific stroke models may help contextualize the unexpected divergence observed between major clinical trials, specifically, the early termination of OCEANIC-AF due to inferior antithrombotic efficacy compared to apixaban versus the positive outcomes in OCEANIC-STROKE for secondary noncardioembolic stroke prevention. While systemic and cardiac models established FXI as a key amplifier of thrombus propagation [32], they poorly captured the distinct pathophysiological boundaries between low-shear cardioembolic thrombus formation in atrial fibrillation [62], which remains heavily dependent on common pathway thrombin generation, and localized, platelet-driven thromboinflammation in focal cerebral ischemia [63, 64]. Consequently, the paucity of disease-specific animal data created a critical gap in anticipating how FXI blockade would perform across distinct vascular beds and clinical indications.

Furthermore, even where stroke-specific preclinical data are available, significant translational constraints inherent to experimental stroke models complicate the bridge from bench to bedside.

A critical appraisal of the preclinical literature reveals several methodological constraints that limit the direct translational validity of contact pathway inhibition in acute stroke. The underlying experimental evidence relies predominantly on transient filament MCAO models in young, healthy male rodents utilizing prophylactic dosing regimens, with a striking scarcity of data incorporating clinical comorbidities, such as hypertension, diabetes, or advanced age, or evaluating safety and efficacy alongside standard reperfusion therapies [65]. To increase translationality, our group demonstrated that C1-esterase inhibitor retains therapeutic neuroprotection when evaluated across extended post-ischemic time windows, female and aged animals, and two distinct species [16]. Moving forward, systematically addressing these experimental constraints through post-reperfusion administration, combination strategies with thrombolysis or thrombectomy, and comorbid stroke models will be crucial to refine the translational pipeline for FXI and FXII targeting.

FXI and FXIa inhibition has transitioned from a promising concept into large-scale human trials, showing potential in secondary noncardioembolic stroke, though variable trial outcomes highlight that efficacy cannot be generalized across all clinical indications. Meanwhile, FXII inhibition offers a strong preclinical rationale and safety profile in stroke models, but its clinical translation lags significantly behind. Moving forward, the therapeutic success of contact pathway inhibitors will rely on addressing these translational gaps, compound-specific verification, and careful patient selection tailored to distinct vascular pathophysiologies.

## Data Availability

No datasets were generated or analysed during the current study.
